# Risk factors for esophageal anastomotic stricture after esophagectomy: a meta-analysis

**DOI:** 10.1186/s12885-024-12625-8

**Published:** 2024-07-19

**Authors:** Yuan Zhong, Ruijuan Sun, Wei Li, Weiqian Wang, Jianpeng Che, Linlin Ji, Bingrong Guo, Chunbo Zhai

**Affiliations:** 1 Department of Thoracic Surgery, Weifang People’s Hospital, Weifang, Shandong China; 2 School of Nursing, Shandong Second Medical University, Weifang, Shandong China; 3 School of Clinical Medicine, Shandong Second Medical University, Weifang, Shandong China

**Keywords:** Risk factors, Anastomotic stricture, Esophagectomy, Meta-analysis

## Abstract

**Background:**

The aim of this study was to assess the risk factors for anastomotic stricture in esophageal cancer patients undergoing esophagectomy. Esophageal anastomotic stricture is the most common long-term complication for esophagectomy. The risk factors for esophageal anastomotic stricture still remain controversial.

**Methods:**

MEDLINE, Cochrane Library, and EMBASE were searched to identify observational studies reporting the risk factors for esophageal anastomotic stricture after esophagectomy. A meta-analysis was conducted to investigate the impact of various risk factors on esophageal anastomotic stricture. The GRADE [Grading of Recommendations Assessment, Development and Evaluation] approach was used for quality assessment of evidence on outcome levels.

**Results:**

This review included 14 studies evaluating 5987 patients.The meta-analysis found that anastomotic leakage (odds ratio [OR]: 2.75; 95% confidence interval[CI]:2.16–3.49), cardiovascular disease [OR:1.62; 95% CI: 1.22–2.16],diabete [OR: 1.62; 95% CI: 1.20–2.19] may be risk factors for esophageal anastomotic stricture.There were no association between neoadjuvant therapy [OR: 0.78; 95% CI:0.62–0.97], wide gastric conduit [OR:0.98; 95% CI: 0.37–2.56],mechanical anastomosis [OR: 0.84; 95% CI:0.47–1.48],colonic interposition[OR:0.20; 95% CI: 0.12–0.35],and transhiatal approach[OR:1.16; 95% CI:0.81–1.64],with the risk of esophageal anastomotic stricture.

**Conclusions:**

This meta-analysis provides some evidence that anastomotic leakage,cardiovascular disease and diabete may be associated with higher rates of esophageal anastomotic stricture.Knowledge about those risk factors may influence treatment and procedure-related decisions,and possibly reduce the anastomotic stricture rate.

**Supplementary Information:**

The online version contains supplementary material available at 10.1186/s12885-024-12625-8.

## Introduction

Esophageal cancer is the eighth most common malignancy in the world, with more than 570 000 new cases diagnosed each year [1]. In China, the incidence of esophageal cancer is the highest in the world, especially in the case of esophageal squamous cell carcinoma, which has been steadily increasing in recent decades [2]. China accounts for more than half of the total number of new cases of esophageal cancer in the world each year. The main treatment for esophageal cancer remains esophagectomy, with a five-year survival rate of around 50% [3]. Despite many improvements in treatment and perioperative care, esophagectomy is still associated with relatively high morbidity and mortality.

Complications of anastomosis are common with esophageal anastomotic stricture being one of the recognised complications affecting 0.5% to 42% of patients following esophageal anastomosis [4–8]. The wide range of reported incidence may be due to the different definition of stricture [4, 5, 7–29]. In this study, anastomotic stricture was defined as follows. Tumor recurrence at the anastomosis site was excluded after esophagectomy. The diameter of the stricture was less than 1 cm under endoscopy, or the conventional type of endoscope (about 1 cm in diameter) could not pass through, accompanied by different degrees of dysphagia.

Patients with esophageal anastomotic stricture had a significantly higher recurrence rate than patients without esophageal anastomotic stricture [30]. Therefore, esophageal anastomotic stricture should be evaluated separately from other complications. The risk factors for esophageal anastomotic stricture still remain controversial. Some of the risk factors associated with a higher incidence of esophageal anastomotic stricture include: Anastomotic leakage [4, 7–9, 12, 13], neoadjuvant therapy [4, 7–9], cardiovascular disease [4, 7–9, 13], diabete [12], colonic interposition [4, 31], wide gastric conduit [10, 12], mechanical anastomosis [11], female and distal location of strictures [30], American society of Aneshesiologists (ASA) grade, cervical anastomosis and transhiatal resections [13]. However, other studies have found that some of these factors are not associated with esophageal anastomotic stricture [7, 8, 31–33]. Therefore, it is important to identify the risk factors for esophageal anastomotic stricture to determine appropriate treatment strategies. Knowledge of these risk factors may help to tailor treatment to each individual patient.

## Methods

### Search strategy

**Fig. 1:**
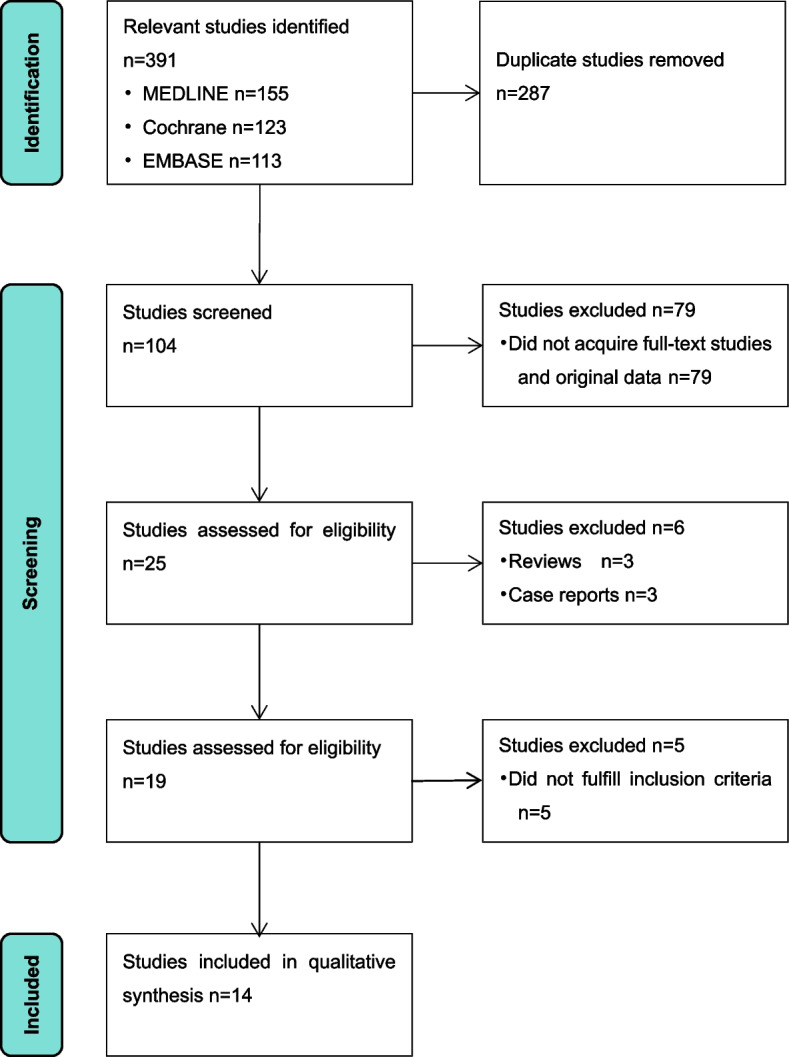
The study selection process

The MEDLINE and EMBASE and Cochrane Library databases were searched [from inception to June 20, 2024].For MEDLINE,eligible trials used the followed medical subject heading (MeSH) terms and search formula:"anastomosis, surgical" [MeSH Terms] AND (stricture [tw] OR stenosis [tw] OR “benign strictures” [tw]) AND esophagectomy [MeSH Terms] AND "Risk Factors" [Title]. The searches were limited to articles published in English. Individually fitted search strategies with similar search terms were also performed in the EMBASE.Only published journal studies were included; unpublished data were not sought.The ‘related articles’ function from MEDLINE was used to broaden the search, and reference lists of included studies were searched for additional relevant studies. Manual searching of reference lists then identified further potentially relevant studies.For the study selection process, see Fig. 1.

### Inclusion criteria

The inclusion criteria for the selected studies as follow.

1. Studies had to report risk factors for esophageal anastomotic stricture in patients who underwent esophagectomy.
2. Anastomotic stricture must have met postoperative dysphagia requiring endoscopic dilatation [34, 35].
3. Anastomotic stricture was benign rather than tumor recurrence.
4. Multivariate regression analysis had to be used for the analysis of risk factors to reduce the risk of confounding in observational studies.

### Quality assessment

The Newcastle–Ottawa scale [36] was used to assess the quality of the studies included. Studies that received seven stars or more were considered to be of higher quality.

The GRADE [Grading of Recommendations Assessment, Development and Evaluation] method was used to assess the quality of evidence at the level of the analytical results [37]. Quality can be rated as high, moderate, low or very low. The GRADE assessment was carried out using GRADEpro software, version 3.6.1 for Windows.

### Data extraction

The following data were extracted from all eligible studies: first author, year of publication, country of origin, study design, number of subjects and risk factors associated with esophageal anastomotic stricture. For risk factors, the focus was on factors previously identified as independent factors, including: anastomotic leakage, cardiovascular disease, diabetes, neoadjuvant therapy, mechanical anastomosis, wide gastric conduit, colonic interposition and transhiatal approach.

### Statistical analysis

The meta-analysis was conducted in accordance with the Preferred Reporting Items for Systematic Reviews and meta-analysis [PRISMA] statement [38]. The odds ratio [OR] was used as a statistical indicator of dichotomous results. ORs were calculated from the original data and presented with 95% confidence intervals [CIs]. For the effect of each variable on the incidence of esophageal anastomotic stricture, the combined odds ratios were calculated. Pooled outcome measures were determined using random-effects models as described by Der Simonian and Laird [39].

Heterogeneity was quantified using I [2]. Slight heterogeneity can account for less than 25% of the variance in point estimates, while significant heterogeneity can account for more than 50% [40].

Statistical analysis was performed using STATA software, version 12.0 [Stata Corporation, College Station, TX, USA].

## Results

### Search results

Our pre-defined search strategy identified 391 studies.Duplicate studies were excluded. Studies that were not available in full text and original data were also excluded. The remaining 25 studies were searched for full-text articles. Three reviews and three case reports were excluded. In total, 19 studies were identified for further analysis. Five of these studies did not meet the inclusion criteria and were excluded from further analysis. Thus, 14 studies that met the inclusion criteria were included in the final meta-analysis. A total of 5987 patients were included. And the overall population was 1571 patients with anastomotic stricture. The overall proportion of anastomotic stenosis in the total study population was 26.2%.

### Quality assessment

The included studies were assessed for risk of bias using the Newcastle–Ottawa scale. The mean score for all studies was 7.6 [range: 7–8]. For most studies, the risk of bias was low and the quality of outcome assessment was good (Table 1).

**Table 1:** Characteristics of the 14 studies included in the meta-analysis to assess the risk of esophageal anastomotic stricture after esophagectomy

Source	Study design	No. of patients	No. of patients of anastomotic stricture	Mean age	Sex (male ratio)	Risk factors	Newcastle–Ottawa scale
Mark van Heijl et al. 2010 Netherlands [4]	Case–control	605	253 (41.7%)	63 [30–85]	76.3	Anastomotic leakage, cardiovascular diseases, colonic interposition	8
P. Honkoop et al. 1996 Netherlands [7]	Case–control	269	114 (42%)	61 [35–82]	74.7	Anastomotic leakage, cardiovascular diseases, Mechanical anastomosis	8
John W Briel et al. 2004 USA [16]	Case–control	393	80 (22%)	NA	NA	Preoperative weight, conduit ischemia, anastomotic leak	8
Haoyao Jiang et al. 2021 China [15]	Case–control	1178	335 (28.4%)	65 [60–69]	82.2	Anastomotic leak	7
Renol M. Koshy et al. 2022 UK [10]	Case–control	705	192 (27.2%)	66 [61–72]	72.9	Anastomotic leakage, wide gastric conduit	8
Katsunori Nishikawa et al. 2020 Japan [11]	Case–control	213	53 (25%)	67 [37–83]	81.1	Triangular anastomotic technique, neoadjuvant therapy, mucosal degeneration	8
Takahiro Hosoi et al. 2019 Japan [12]	Case–control	263	90 (38%)	65 [40–86]	81.8	Chronic obstructive pulmonary disease, Anastomotic leakage, narrow gastric conduit	8
Zuhair Ahmed et al. 2017 Ireland [13]	Case–control	524	125 (24.5)	62 [51–73]	73.9	Cervical reconstruction, transhiatal approach, cardiovascular diseases	8
R. P. Sutcliffe et al. 2008 UK [8]	Cohort	177	48 (27%)	63 [54–72]	69.7	Cervical reconstruction, delayed gastric emptying	8
Leonie Haverkamp et al. 2013 Netherlands [27]	Case–control	390	137 (35%)	63 [52–71]	72.8	Cervical reconstruction	7
Robert Tyler et al. 2019 UK [28]	Case–control	154	15 (10%)	64 [54–74]	77.3	Anastomotic leak	7
Koji Tanaka et al. 2018 Japan [5]	Case–control	213	29 (13.6%)	69 [63–75]	86.2	Location (upper part of the esophagus), cardiovascular diseases, anastomotic leak	8
Yi-Min Gu et al. 2019 China [29]	Case–control	702	62 (8.8%)	58 [52–63]	80.6	Cervical reconstruction, hypertension	7
Dong-shan Zhu et al. 2020 China [14]	Case–control	201	38 (18.9%)	64 [45–80]	66.2	Wide gastric conduit	7

*NA* Not available

The GRADE method was used to assess the quality of the evidence at the level of the analysis results. The quality of the evidence was assessed as high, medium or low for these risk factors (Table 2).

**Table 2:** Summary finding of risk factors eligible for meta-analysis

Risk factor	Number of patients/studies	Regarded a risk factor	Pooled odds ratio	Quality of evidence [GRADE]
Anastomotic leakage	5257/11	Yes	2.75 [95% CI: 2.16–3.49]	⊕⊕⊕⊕⊕ High
Neoadjuvant therapy	3507/7	No	0.78 [95% CI: 0.62–0.97]	⊕⊕⊕⊕○ Moderate
Cardiovascular disease	2362/7	Yes	1.62 [95% CI: 1.22–2.16]	⊕⊕⊕⊕⊕ High
Diabete	3827/7	Yes	1.62 [95% CI: 1.20–2.19]	⊕⊕⊕⊕⊕ High
Wide gastric conduit	2367/4	No	0.98 [95% CI: 0.37–2.56]	⊕⊕⊕○○ Low
mechanical anastomosis	2756/4	No	0.84 [95% CI: 0.47–1.48]	⊕⊕⊕○○ Low
Colonic interposition	1183/3	No	0.20 [95% CI: 0.12–0.35]	⊕⊕⊕⊕⊕ High
Transhiatal approach	1525/3	No	1.16 [95% CI: 0.81–1.64]	⊕⊕⊕⊕⊕ High

GRADE Working Group grades of evidence:

High quality: Further research is very unlikely to change our confidence in the estimate of effect

Moderate quality: Further research is likely to have an important impact on our confidence in the estimate of effect and may change the estimate

Low quality: Further research is very likely to have an important impact on our confidence in the estimate of effect and is likely to change the estimate

Very low quality: We are very uncertain about the estimate

### Risk factors for esophageal anastomotic stricture

#### Anastomotic leakage

**Fig. 2:**
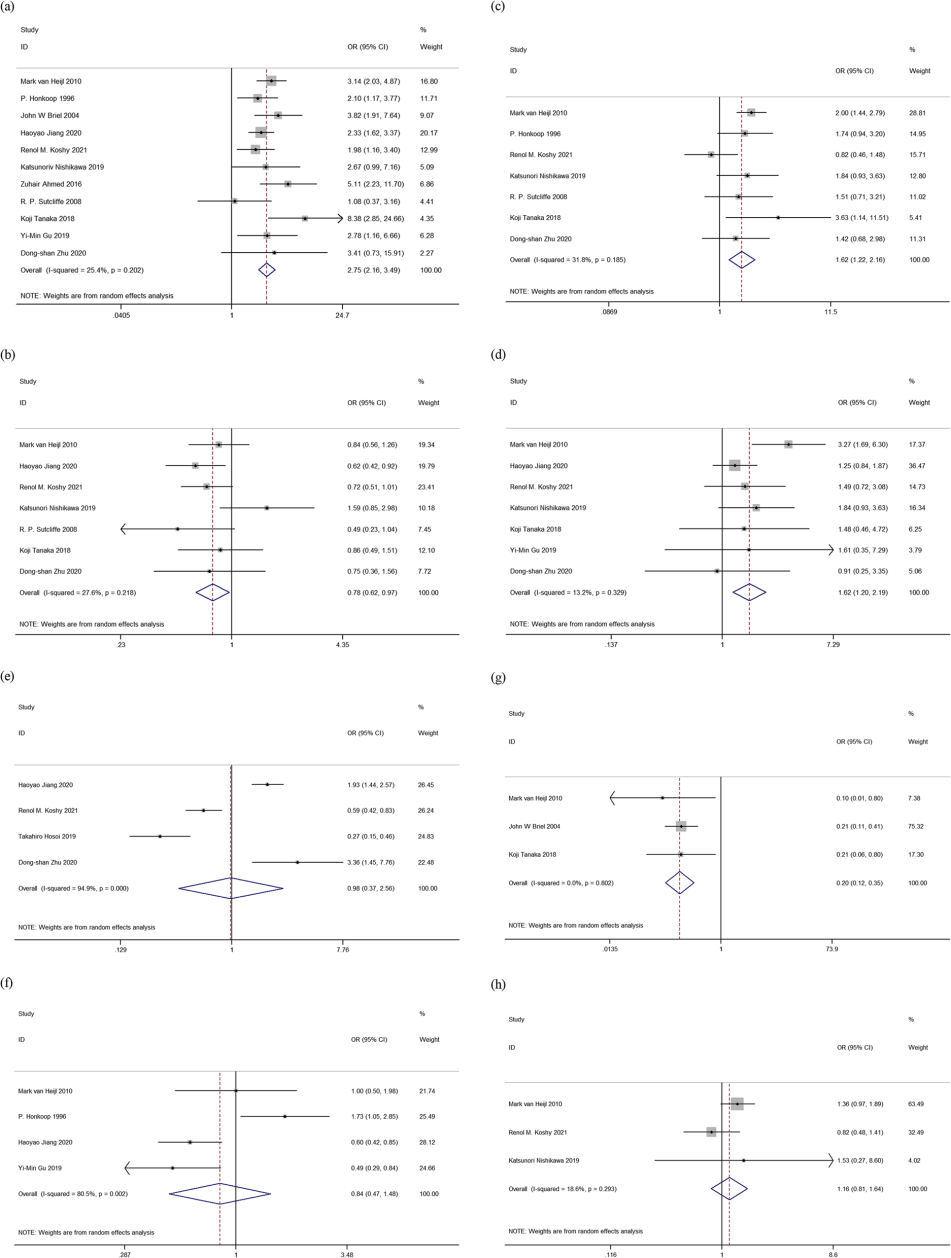
Forest plots for risk factor eligible for meta-analysis. **a** Anastomotic leakage. **b** Neoadjuvant therapy. **c** Cardiovascular disease. **d** Diabete. **e** Wide gastric conduit. **f** Mechanical anastomosis. **g** Colonic interposition. **h** Transhiatal approach

Total 11 studies [4, 5, 7, 8, 10, 11, 13–16, 29] [*n* = 5257] assessed anastomotic leakage as a risk factor. A meta-analysis of these studies using a random-effects model found an overall OR of 2.75 [95% CI: 2.16–3.49], indicating that anastomotic leakage was associated with a high risk of esophageal anastomotic stricture (Fig. 2a). Heterogeneity between studies was mild [*p* = 0.202; *I*^2^ = 25.4%] and using funnel plots and Egger's test [*p* = 0.408], there was no evidence of significant publication bias. Using the GRADE method, the quality of the evidence was judged to be high.

#### Neoadjuvant treatment

Seven studies [4, 5, 8, 10, 11, 14, 15] [*n* = 3507] of these trials found an overall OR of 0.78 [95% CI: 0.62–0.97] in a random-effects model, suggesting that neoadjuvant treatment did not increase the risk of esophageal anastomotic stricture (Fig. 2b). Heterogeneity between studies was mild [*p* = 0.218; *I*^2^ = 27.6%] and using funnel plots and Egger's test [*p* = 0.665], there was no evidence of significant publication bias. Using the GRADE approach, the quality of the evidence was assessed as moderate.

#### Cardiovascular diseases

Seven studies [4, 5, 7, 8, 10, 11, 14] [*n* = 2362] assessed cardiovascular disease as a risk factor. A meta-analysis of these studies using a random-effects model found an overall OR of 1.62 [95% CI: 1.22–2.16], indicating that cardiovascular disease was associated with the risk of esophageal anastomotic stricture (Fig. 2c). Heterogeneity between studies was mild [*p* = 0.185; *I*^2^ = 31.8%] and no significant evidence of publication bias was found using the funnel plot and Egger's test [*p* = 0.822]. The quality of the evidence was assessed as high using the GRADE method.

#### Diabete

Seventh studies [4, 5, 10, 11, 14, 15, 29] [*n* = 3827] assessed diabete as a risk factor. In meta-analysis of these studies using a random-effects model, an overall OR of 1.62 [95% CI: 1.20–2.19] was found, suggesting that diabetes was a risk factor for esophageal anastomotic stricture (Fig. 2d). Heterogeneity between studies was low [*p* = 0.329; *I*^2^ = 13.2%], and there was no significant evidence of publication bias using the funnel plot and Egger's test [*p* = 0.829]. The quality of the evidence was rated high using the GRADE approach.

#### Wide gastric conduit

Only four studies [10, 12, 14, 15] [*n* = 2367] evaluated wide gastric conduit as a risk factor. A meta-analysis of these studies using a random-effects model found an overall OR of 0.98 [95% CI: 0.37–2.56], indicating that wide gastric conduit was not a risk factor for esophageal anastomotic stricture (Fig. 2e). However, the heterogeneity was very high [*p* = 0.000; *I*^2^ = 94.9%]. The results were not reliable. The quality of the evidence was regarded as low based on the GRADE approach.

#### Mechanical anastomosis

Four studies [4, 7, 15, 29] [*n* = 2756] evaluated the effect of anastomosis techniques. A meta-analysis of these studies using a random-effects model showed an overall OR of 0.84 [95% CI: 0.47–1.48], indicating that mechanical anastomosis was not a risk factor for esophageal anastomotic stricture (Fig. 2f). Heterogeneity was, however, very high [*p* = 0.002; *I*^2^ = 80.5%]. The results were not reliable. Based on the GRADE approach, the quality of the evidence was assessed as low.

#### Colonic interposition

Colonic interposition was assessed as a risk factor in only three studies [4, 5, 16] [*n* = 1183]. In meta-analysis of these studies using a random-effects model, an overall OR of 0.20 [95% CI: 0.12–0.35] was found, suggesting that colonic interposition was not a risk factor for esophageal anastomotic stricture (Fig. 2g). There was no heterogeneity between studies [*p* = 0.802; *I*^2^ = 0.0%], and no significant evidence of publication bias was found using funnel plot and Egger's test [*p* = 0.401]. The GRADE approach was used to assess the quality of the evidence as high.

#### Transhiatal approach

Only three studies [4, 10, 11] [*n* = 1525] evaluated transhiatal approach as a risk factor. A meta-analysis of these studies using a random effects model found an overall OR of 1.16 [95% CI: 0.81–1.64], suggesting that transhiatal approach was not a risk factor for stricture of the esophageal anastomosis (Fig. 2h). Study heterogeneity was low [*p* = 0.293; *I*^2^ = 18.6%] and there was no significant evidence of publication bias by funnel plot and Egger's test [*p* = 0.856]. The quality of evidence was assessed as high using the GRADE method.

## Discussion

This is the first meta-analysis of observational studies designed to assess risk factors for esophageal anastomotic stricture after esophagectomy. The meta-analysis showed that anastomotic leakage, cardiovascular disease and diabete are associated with esophageal anastomotic stricture. No association was found between neoadjuvant therapy, wide gastric conduit, mechanical anastomosis, colonic interposition and transhiatal approach with the risk of esophageal anastomotic stricture. Based on the results of the meta-analysis of GRADE approaches, the quality of the evidence was assessed as low to high. The studies included in the meta-analysis were judged by the NOS to be of good quality for the assessment of outcomes. Thus, the low quality of the results was not due to the bias of the studies, but mainly to the observational nature of the studies, which was initially rated as low by GRADE.

In this meta-analysis, anastomotic leakage is an important risk factor for anastomotic stricture. The transition of anastomotic leakage to a later benign stricture, although not fully investigated, may be associated with concomitant fibrosis leading to scar formation and subsequent stricture formation. This secondary healing process results in strictures that are more likely to be refractory [11]. Low oxygen supply and poor perfusion in the anastomosis, which are likely to be the cause of leakage, also lead directly to the formation of anastomotic strictures [4]. The local infection caused by anastomotic leakage, as well as repetitive trauma and stimulus, can promote fibrous tissue proliferation and scar formation, leading to gradual narrowing of the anastomosis [11, 41, 42].

In this meta-analysis, neoadjuvant treatment did not increase the risk of anastomotic stricture. Interestingly, patients who received neoadjuvant chemotherapy were less likely to develop stricture. Prior chemotherapy reduced the likelihood of anastomotic stricture by almost 70% [30]. One hypothesis is that the phenomenon of a low inflammatory response to foreign bodies, based on the patient's compromised immune system, may lead to a lower incidence of anastomotic strictures due to the foreign body response [30, 43]. Another hypothesis is that chemotherapy reduces the postoperative inflammatory response (fibrosis and intimal hyperplasia) leading to luminal stricturing [30, 44–46].

The meta-analysis found that a history of cardiovascular disease is an important risk factor for anastomotic stricture [7, 33]. A leakage at the anastomosis site can lead to local ischemia and hypoxia, which can progress to anastomotic stenosis. Similarly, postoperative gastric tube anastomosis circulate has already been damaged, and patients with a history of cardiovascular disease will exacerbate this insufficient circulate and further worsen the condition [47]. Although significant atherosclerosis is rarely observed in the right gastroepiploic artery, it is possible that some degree of luminal stenosis may be sufficient to cause conduit ischaemia if accompanied by impaired oxygen exchange or any cardiovascular or hemodynamic factors affecting perfusion [48]. Taken together, these findings suggest that arterial atherosclerosis or microvascular stenosis may be sufficient to cause conduction hypoperfusion in the presence of oxygen exchange disturbances or cardiovascular or hemodynamic factors affecting systemic circulation.

Diabete has also been shown to increase the incidence of anastomotic stricture after esophagectomy. Its aetiology is similar to that of cardiovascular disease. Diabete can lead to atherosclerosis and microcirculatory disorders. Blood supply to the proximal part of the gastric conduit depends on intragastric collateral flow and microvascular perfusion. If diabete causes hyalinosis and microcirculatory disturbances, ischaemia of this part of the duct may occur [31, 49]. Therefore, it is important to monitor the patient's blood glucose levels to maintain uninterrupted microcirculation. Recent studies suggest that anastomosis reconstruction 4–5 days after vascular dissection may promote neovascularisation and prevent anastomosis-related complications [31, 50, 51].

It is not clear from the meta-analysis what the effect of wide gastric conduit is on anastomotic stricture. In some studies, an extensive gastric conduit was associated with high risk [14, 15, 52–54], but not in others [10, 11, 55–58]. In contrast to the wide gastric conduit, the diameter of the narrow band is similar to the diameter of the natural esophagus. The narrow conduit allowed the greater curvature to be extended as much as possible, which reduces the strain of the anastomosis and allows the anastomosis to be performed close to an area where the blood supply from the right gastroepiploic artery is intact [15].

Mechanical anastomosis instead of hand-sewn anastomosis accelerating anastomotic strictures are not well-defined. A common endoscopic finding in patients with esophagogastric anastomosis is the presence of protruding sutures and/or staples in the esophageal lumen. The presence of a foreign body may promote inflammation and scarring, which complicates treatment [44]. Differences in the incidence of strictures between manually sutured and stapled anastomoses may be due to inadequate attachment of the mucosa to the staples [59].

In this meta-analysis, colonic interposition did not increase the risk of stricture. Anastomotic stricture are more frequent after gastric reconstruction. The blood supply to the colon used for colonic interposition is completely vascularized by arteries, which may explain the relatively large difference in the incidence of stricture [4, 32, 60]. There's another explanation that gastric pull-up suggests that reflux of gastric juices may contribute to stricture formation [16]. The observation that late stricture (more than 1 year) occurred only in patients with gastric pull-up additionally supports a reflux etiology.

The transhiatal approach was not an independent risk factor for anastomotic stricture. During transhiatal esophagectomy, the esophagus was removed directly through an extended diaphragmatic hiatus in a straight line of sight to the lower pulmonary vein. After mobilization of the right or left esophagus, the intrathoracic esophagus was squatted proximally to distally using a vein cutter [4]. The transthoracic approach was similar to the transhiatal approach, as was the creation of a 3 to 4 cm wide gastric tube. In a meta-analysis, the transhiatal approach was not statistically associated with the number of anastomotic complications [49, 50, 61].

The present meta-analysis has some limitations: the risk factor for esophageal anastomotic stricture can only be examined in observational studies, which poses a risk of bias that cannot be eliminated by a tailored analysis. Observational studies were considered to be of lower quality than randomised controlled trials, so the conclusions we present are based on studies of lower quality. The included studies differed in terms of risk factors for disease severity, location and duration, surgical indication and type of surgical procedure. We therefore used random-effects models in the meta-analysis, which provided more conservative conclusions in the presence of heterogeneity. For observational studies, we sought to reduce the risk of confounding factors by selecting studies using multivariate regression. However, without including all known and unknown confounders, this risk cannot be completely eliminated.

In conclusion, the prevalence of anastomotic leakage, cardiovascular disease and diabetes was associated with a higher incidence of esophageal anastomotic stricture in patients undergoing esophagectomy. This may help surgeons to better decide on appropriate treatment strategies for individual patients.

### Supplementary Information

Supplementary Material 1.

## Data Availability

Data is provided within the manuscript or supplementary information files.
