# Supplementary figures and images for: Risk factors for esophageal anastomotic stricture after esophagectomy: a meta-analysis
Source: BMC Cancer. 2024 Jul 19;24:872. doi: 10.1186/s12885-024-12625-8 (PMC11264988; doi:10.1186/s12885-024-12625-8)

(a)


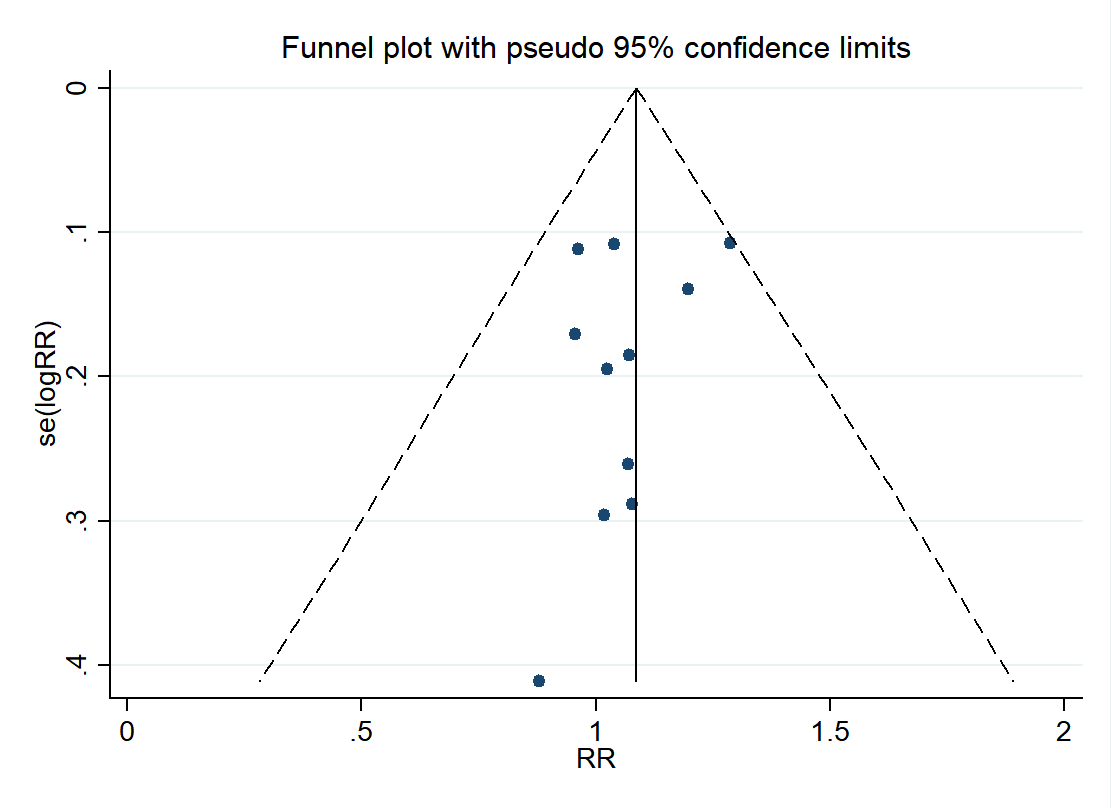


(b)


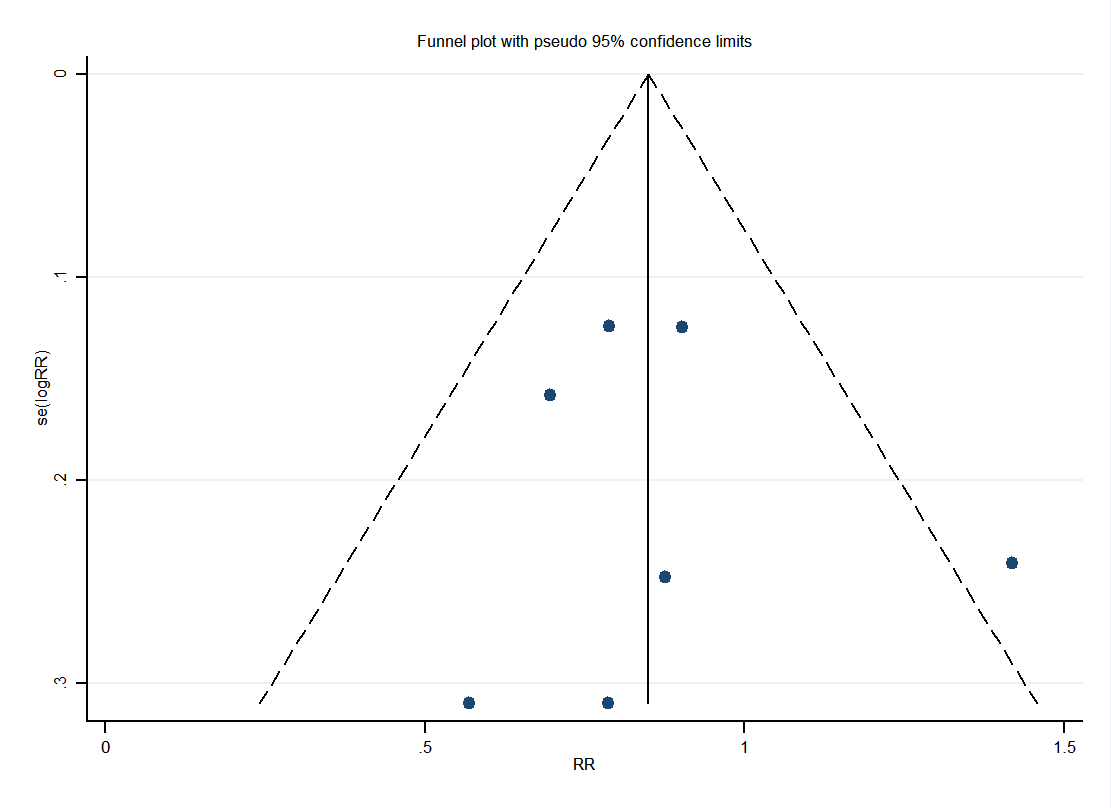


(c)


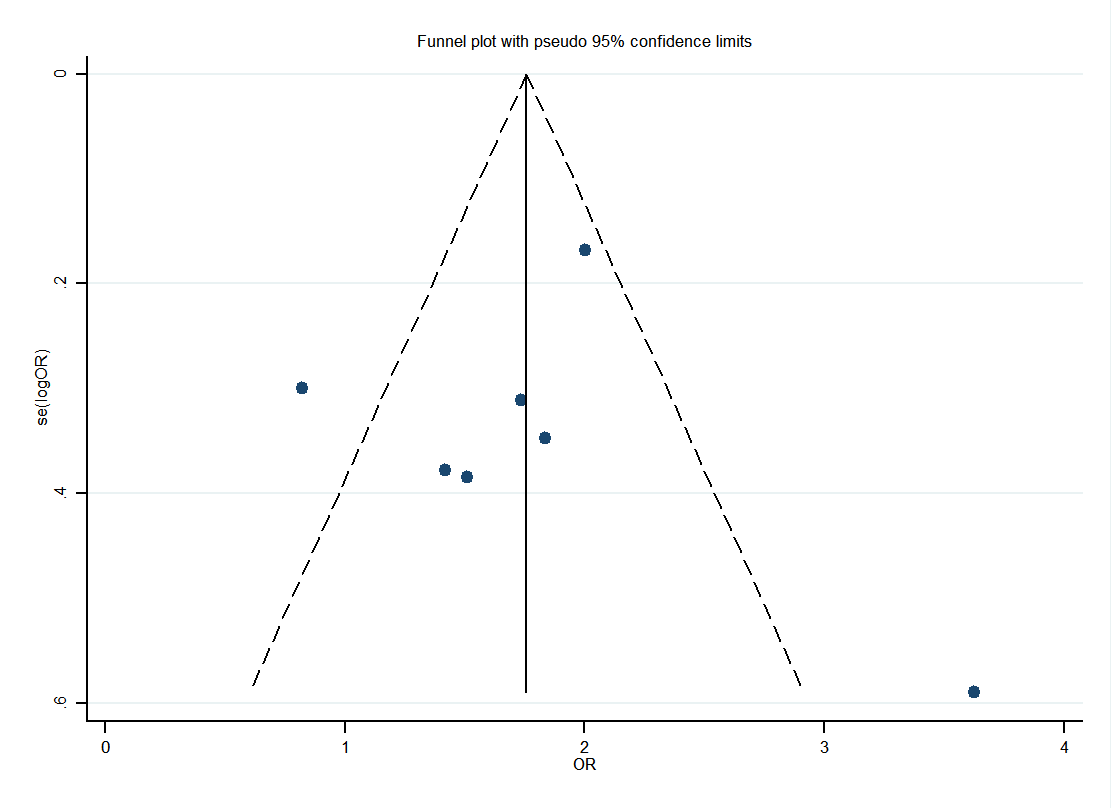


(d)


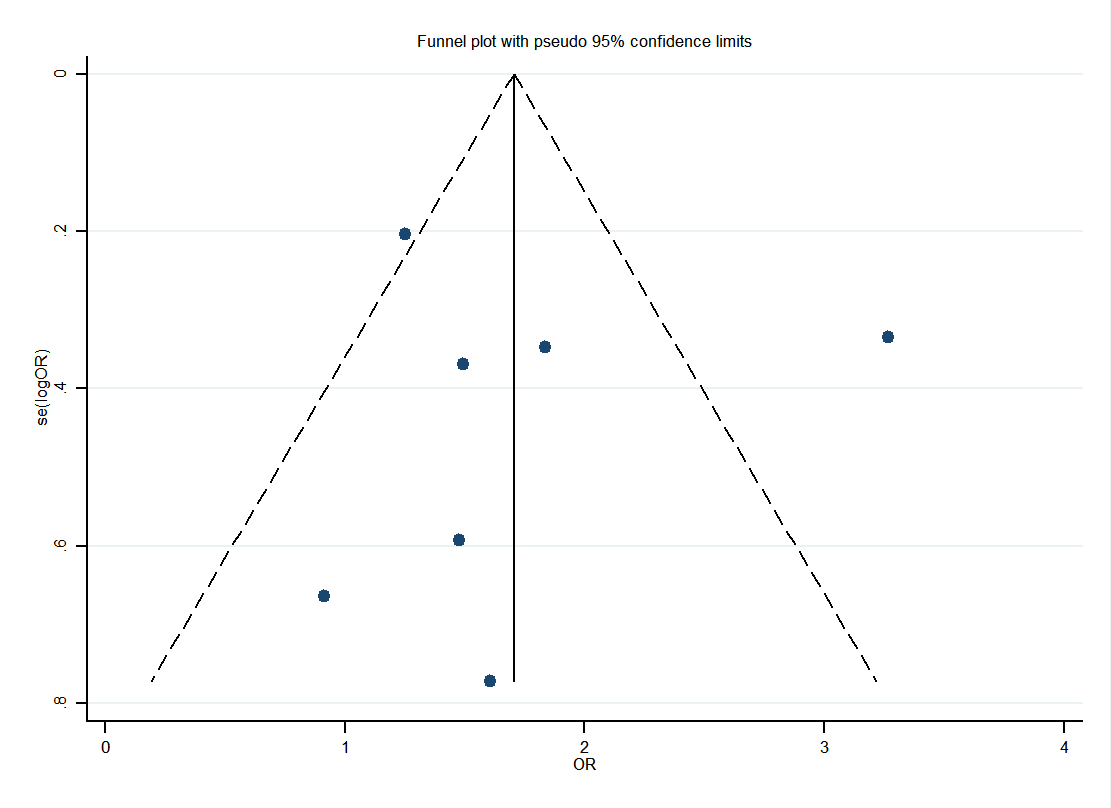


(a)Anastomotic leakage. (b)Neoadjuvant therapy. (c) Cardiovascular disease. (d)Diabete.

Supplement: Supplementary file 1 — Supplementary Material 1. [file 12885_2024_12625_MOESM1_ESM.zip › funnel plots 2024-07-06.docx]
