# Supplementary material for: Risk factors for esophageal anastomotic stricture after esophagectomy: a meta-analysis
Source: BMC Cancer. 2024 Jul 19;24:872. doi: 10.1186/s12885-024-12625-8 (PMC11264988; doi:10.1186/s12885-024-12625-8)
Supplement: Supplementary file 1 — Supplementary Material 1. [file 12885_2024_12625_MOESM1_ESM.zip › Egger's test.doc]

(a)

(b)

(c)

(d)

(e)

(f)

Figure.Egger's test for risk factor eligible for meta-analysis. (a)Anastomotic leakage. (b)Neoadjuvant therapy. (c)Cardiovascular disease. (d)Diabete. (e)Colonic interposition. (f)Transhiatal approach.
